# Event-Based Machine Vision for Edge AI Computing

**DOI:** 10.3390/s26030935

**Published:** 2026-02-01

**Authors:** Paul K. J. Park, Junseok Kim, Juhyun Ko, Yeoungjin Chang

**Affiliations:** 1 Samsung Electronics, Hwaseong 18448, Republic of Korea; paulpark@gachon.ac.kr (P.K.J.P.); junseok7.kim@samsung.com (J.K.); juhyun03.ko@samsung.com (J.K.); 2 Department of Semiconductor Display, Gachon University, Seongnam 13120, Republic of Korea

**Keywords:** dynamic vision sensor, event-based vision, edge AI, neuromorphic, timestamp-based encoding, polarity-agnostic event representation, home occupancy sensing, human detection, human pose estimation, hand posture recognition

## Abstract

**Highlights:**

**What are the main findings?**

- We present an event image representation that preserves moving-edge structure while reducing data volume for downstream processing.
- The proposed event-based edge AI computing achieves an 11× speed-up for human detection and pose estimation.

**What are the implications of the main findings?**

- The approach enables privacy-friendly, always-on home occupancy sensing under tight edge constraints.
- Combining event encoding with compact models is an effective deployment recipe for motion-centric edge AI tasks.

**Abstract:**

Event-based sensors provide sparse, motion-centric measurements that can reduce data bandwidth and enable always-on perception on resource-constrained edge devices. This paper presents an event-based machine vision framework for smart-home AIoT that couples a Dynamic Vision Sensor (DVS) with compute-efficient algorithms for (i) human/object detection, (ii) 2D human pose estimation, (iii) hand posture recognition for human–machine interfaces. The main methodological contributions are timestamp-based, polarity-agnostic recency encoding that preserves moving-edge structure while suppressing static background, and task-specific network optimizations (architectural reduction and mixed-bit quantization) tailored to sparse event images. With a fixed downstream network, the recency encoding improves action recognition accuracy over temporal accumulation (0.908 vs. 0.896). In a 24 h indoor monitoring experiment (640 × 480), the raw DVS stream is about 30× smaller than conventional CMOS video and remains about 5× smaller after standard compression. For human detection, the optimized event processing reduces computation from 5.8 G to 81 M FLOPs and runtime from 172 ms to 15 ms (more than 11× speed-up). For pose estimation, a pruned HRNet reduces model size from 127 MB to 19 MB and inference time from 70 ms to 6 ms on an NVIDIA Titan X while maintaining a comparable accuracy (mAP from 0.95 to 0.94) on MS COCO 2017 using synthetic event streams generated by an event simulator. For hand posture recognition, a compact CNN achieves 99.19% recall and 0.0926% FAR with 14.31 ms latency on a single i5-4590 CPU core using 10-frame sequence voting. These results indicate that event-based sensing combined with lightweight inference is a practical approach to privacy-friendly, real-time perception under strict edge constraints.

## 1. Introduction

Recently, there has been an increasing demand for the development of energy-efficient sensing and computing techniques in intelligent edge devices for smart-home/IoT applications [1]. Conventional precise machine vision tasks such as biometric technologies based on face and iris recognition need high-resolution image sensors and deep neural networks to improve performance. These deep neural networks, which require substantial computational resources, can be implemented in high-end smartphones or large-scale computing servers. However, with the advent of Artificial Intelligence of Things (AIoT), it has become necessary to develop pragmatic machine vision tasks such as presence/obstacle detection and gesture/posture recognition which can be deployed on standalone low-end edge devices. In practical AIoT deployments, edge devices are often expected to operate continuously under strict constraints on power consumption, memory capacity, and computation throughput. For this reason, good-enough perception for decision making (e.g., detecting a moving person, counting occupants, tracking body key-points for interaction, recognizing hand postures) becomes more important than producing photo-realistic images. These edge AI computing tasks require uncolored, low-bit, and low-resolution image sensors because computational resources are insufficient to handle a large volume of information. In addition, the system-level overhead of capturing and storing dense video streams can dominate both device cost and service cost, especially in always-on home surveillance and smart-home monitoring scenarios.

It should be noted that a conventional high-resolution image sensor is appropriate for use in precise and static tasks such as face and object recognition while event-based vision sensors are more suitable for fast and dynamic motion perception. In particular, event-based sensors provide motion-centric information by reporting asynchronous brightness changes in pixels, which naturally suppress static background and can reduce redundant computation in downstream neural processing. This sensing paradigm is therefore attractive for edge AI computing, where the dominant challenges include latency, bandwidth, and memory traffic. From the algorithmic perspective, event streams can also be interpreted as sparse spatiotemporal signals, enabling lightweight feature extraction when the task is closely tied to motion (e.g., surveillance, pose estimation, gestures). Nevertheless, a key practical challenge is that most state-of-the-art vision algorithms and neural networks are designed for frame-based inputs, and edge deployment requires not only efficient sensing but also efficient representations and models. Therefore, in this paper, we propose and demonstrate event-based machine vision techniques such as object detection, human pose estimation, and hand posture recognition, then show how their performances can be optimized in real standalone edge devices. Our goal is to present an end-to-end perspective: (i) how event-based sensing provides advantages in data sparsity and background filtering, (ii) how event data can be converted into a CNN-friendly image-like representation without losing the core temporal cues, (iii) how neural networks can be redesigned (e.g., pruning and quantization) to achieve low-latency inference on constrained platforms while maintaining acceptable accuracy. Section 2 introduces the event-based sensing principle using a Dynamic Vision Sensor (DVS) and discusses why its sparse, quantized outputs are suitable for low-latency edge perception. Section 3 presents the event-to-image conversion method and introduces a polarity-agnostic timestamp-based image generation scheme to obtain clearer representations than simple temporal accumulation. Section 4 describes the event-based object detection task for home surveillance, including region proposal behavior and compute-aware network optimization (stride, reduced layers, and mixed-bit quantization). Section 5 focuses on human pose estimation and explains how a high-performing HRNet backbone can be pruned for DVS inputs while preserving accuracy–latency trade-offs. Section 6 demonstrates hand posture recognition for a human–machine interface (HMI) and validates real-time feasibility on a low-end CPU. Finally, Section 7 summarizes the main findings and outlines the role of computationally efficient event-based processing for future edge AI devices.

The contributions of this paper are summarized as follows:

- Polarity-agnostic recency encoding for edge-centric perception. We introduce a polarity-agnostic timestamp/recency image representation that emphasizes motion-induced edge shape while avoiding polarity-dependent failure cases, making it suitable for occupancy-oriented home surveillance tasks.
- Controlled evaluation with fixed downstream networks. We validate the proposed representation under fixed downstream network and training protocols, isolating the effect of event-to-image encoding from architectural changes, and demonstrate that a competitive recognition performance can be achieved with lightweight models.
- Edge deployment optimization and system-level evaluation (Section 4, Section 5 and Section 6). We present a practical edge computing flow and report end-to-end latency/compute/accuracy trade-offs. The presented pruning/layer reduction, mixed-bit quantization, and stride choices are standard engineering optimizations documented for reproducibility, showing that the proposed method can operate in real time under strict edge constraints.

## 2. Event-Based Sensing

### 2.1. Literature Review

Event-based vision and event cameras have been comprehensively reviewed in several surveys. Gallego et al. provide a foundational overview of event camera sensing principles and core advantages (asynchronous measurements, high temporal resolution, and high dynamic range) and summarize representative processing techniques from low-level vision (e.g., feature tracking and optical flow) to high-level tasks (e.g., recognition and detection), including common event representations and learning-based approaches [2]. More recently, Cimarelli et al. presented a broad review that integrates hardware evolution, algorithmic progress, and real-world applications of neuromorphic/event cameras in a unified structure, while also discussing practical challenges and adoption barriers relevant to deployment [3]. In addition, Cazzato and Bono provide an application-driven survey that organizes event-based computer vision methods by domain and highlights key achievements and open issues across application areas [4]. Finally, Chakravarthi et al. survey recent event camera innovations, including sensor model developments and commonly used datasets/simulators that support benchmarking and system validation [5]. In contrast to these survey papers, which broadly summarize event camera principles and general-purpose algorithms across diverse domains, this work focuses on a deployment-oriented smart-home setting and provides a concrete end-to-end framework—timestamp-based encoding plus edge-optimized inference—for always-on occupancy-related tasks with explicit system-level evidence (data volume, latency, FLOPs/model size, and accuracy).

### 2.2. Frame-Based Sensors vs. Event-Based Sensors

**Figure 1:**
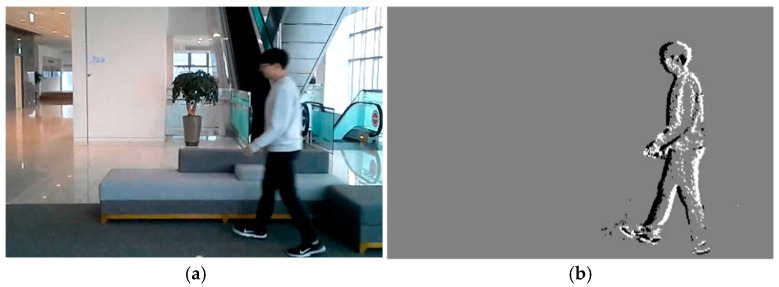
Image examples of (**a**) CIS and (**b**) DVS captured in an indoor environment.

We recently proposed an activity-driven and event-based vision sensor known as DVS [6,7,8]. DVS is a unique image sensor inspired by biological visual systems. Each DVS pixel produces a stream of asynchronous events just as the ganglion cells of biological retina do. By processing the information of local pixels having relative intensity changes instead of entire images at fixed frame rates, the computational requirements can be reduced dramatically. Thanks to sparsity and binary features of DVS images, we expect that the recognition task can also be achieved with low computational cost and latency. Figure 1 shows example images of CMOS Image Sensor (CIS) and DVS captured in an indoor environment. Figure 1b clearly shows that the background information can be filtered out while moving edges are obtained from DVS. Thus, the moving person can be detected simply by capturing the original DVS image. On the other hand, in order to detect the moving person from the CIS image as shown in Figure 1a, extracting the features is required via massive calculations based on convolutional, pooling, and activation layers then classifying them into several objects by utilizing fully connected layers [9].

### 2.3. Data Size Comparison

**Figure 2:**
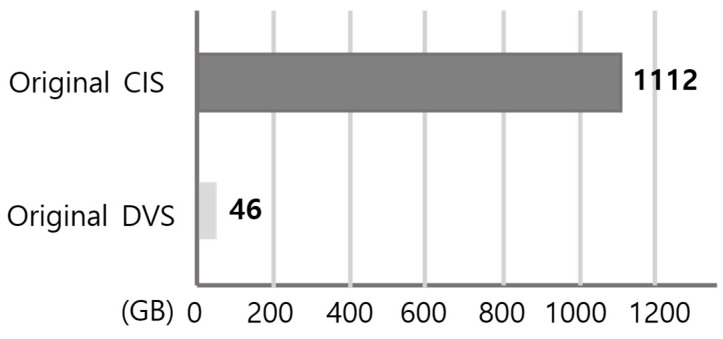
Data size comparison between DVS and CIS (640 × 480) during a 24 h indoor recording scenario with two moving subjects. The recorded raw DVS stream is approximately 30 times smaller than the CIS video with the same duration and resolution, reducing interface and storage requirements.

The image data stored in edge AI devices can be decreased significantly by using DVS rather than CIS. This is mainly because the data obtained from DVS is inherently quantized and sparse, and it is generated only when meaningful intensity changes occur, rather than being sampled as dense frames at a fixed frame rate. Figure 2 compares the recorded raw data size of DVS and CIS under the same spatial resolution (640 × 480) and the same monitoring conditions. In this experiment, two humans repeatedly moved in front of the sensors over a 24 h period, which is representative of real smart-home surveillance where motion occurs intermittently rather than continuously. Because CIS continuously records full frames regardless of activity, its stored data volume scales primarily with the frame rate and recording duration. In contrast, the DVS output is activity-driven and scales with the amount of motion and edge activity in the scene, which is typically sparse in indoor environments. As a result, the total amount of DVS data becomes about 30× smaller than that of CIS in this 24 h scenario, demonstrating that event-based sensing can dramatically reduce the burden of continuous recording. Therefore, the required interface throughput, memory bandwidth, and storage capacity can be remarkably alleviated, enabling a longer retention time and lower system cost for always-on edge AI devices.

**Figure 3:**
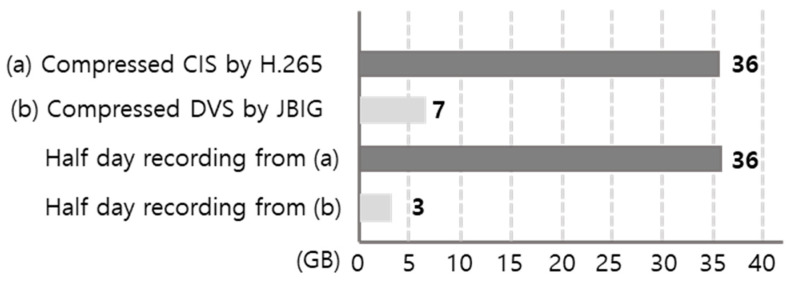
Compressed data volume comparison between CIS and DVS for long-term monitoring (24 h). CIS frames (dense color video) were compressed using the standard H.264/AVC video codec (typically lossy), reflecting realistic surveillance video storage [10]. DVS outputs were converted to non-color, low-bit (bi-level/binary) event images and compressed using JBIG (lossless bi-level image compression) with a fixed encoder configuration [11]. We use modality-appropriate standard codecs because CIS and DVS data have fundamentally different structures (dense color video vs. sparse binary edge activity). Even after compression, the DVS stream remains approximately 5× smaller than the CIS stream in this representative 24 h recording.

Even in the case of data compression, the advantage of DVS remains significant. As shown in Figure 3, the compressed size of DVS data is still about five times smaller than that of CIS. This result indicates that the sparsity of event streams is not only beneficial before compression but also provides a favorable structure for storage after compression because redundant background content and temporally repeated frames are largely absent in DVS recordings. From a system perspective, this sparse feature of DVS data is beneficial to both end customers and service providers. Specifically, it can reduce CAPEX (e.g., local storage provisioning, memory, and interface design margins) and OPEX (e.g., data archival, network transfer, and server-side processing costs) in real deployments that require long-term recording and frequent retrieval. In addition, maintaining a smaller compressed footprint can facilitate scalable deployment across multiple devices in a home/building environment while keeping operational costs manageable.

## 3. Timestamp-Based Image Generation

To use a DVS for vision tasks, the asynchronous event stream must be converted to an image-like input. A common baseline is temporal accumulation, where events are summed within a fixed time window to produce a frame image [12]. While simple, accumulation can be unreliable in edge AI settings with frequent low-motion intervals (e.g., indoor monitoring) because sparse events may yield weak contours and the representation does not explicitly encode how recently a pixel was activated. Recency-based representations address this by storing the latest event timestamp at each pixel (often referred to as a Surface of Active Events, SAE [13]) and mapping the elapsed time to an intensity value. In time surface methods [14,15,16], this mapping is frequently implemented as an exponential decay and is commonly polarity-conditioned. Such polarity separation is useful when one explicitly models signed contrast changes or directional motion cues, but it is not strictly required for edge-shape recognition problems where the goal is to detect the presence and geometry of edges. Thus, in this work, we introduce polarity-agnostic global recency encoding—defined by timestamp-based intensity mapping—that is explicitly tailored to edge intensity tasks (object detection, human pose estimation, and hand posture recognition). Instead of maintaining separate recency maps for ON and OFF events, we update a single per-pixel timestamp memory with any event regardless of polarity. This avoids contour fragmentation when ON/OFF events are imbalanced (e.g., edges that predominantly generate only one polarity under certain motion/lighting conditions) and reduces memory/compute by eliminating multi-channel polarity handling. The key idea is to store the latest timestamp at each pixel and convert the time difference into an intensity value. In other words, each pixel intensity represents the recency of activity rather than the number of events. This approach can preserve important temporal cues while still providing a frame-like representation that can be directly used by conventional Convolutional Neural Network (CNN)-based techniques. More specifically, when an event is generated at a pixel, the current timestamp is updated for that pixel. Let an event be *e_i_* = (*x_i_*, *y_i_*, *t_i_*, *p_i_*), where (*x_i_*, *y_i_*) is the pixel location, *t_i_* is the timestamp, and *p_i_* ∈ {+1, −1} is the polarity. At a sampling time *t_k_*, we define the polarity-agnostic recency surface

(1) Sx,y=maxi:xi,yi=(x,y) ti

i.e., the most recent event time at each pixel regardless of polarity. The per-pixel recency is Δ*t*(*x*, *y*) = *t_k_* − *S*(*x*, *y*). To convert recency into an 8-bit intensity image, we use an exponential mapping with a temporal sensitivity parameter *T_s_* and intensity amplitude *I_max_* as follows:

(2) $$I_{k}\left(x,y\right)=\;\left\{\begin{array}{l} I_{max}exp\left(-\frac{∆t(x,y)}{T_{s}}\right),\;∆t(x,y)\leq T_{s}\\ 0,\;otherwise,\;\;\;\; \end{array}\right.$$

where pixels with more recent events become brighter. In our indoor setup, we set *T_s_* = 100 ms and *I_max_* = 255 for 8-bit grayscale scaling. Empirically, *T_s_* acts as a motion-dependent temporal sensitivity knob. We found that *T_s_* ≈ 20 ms is suitable for near-field, fast hand gesture motion (~1 m), whereas *T_s_* ≈ 100 ms provides the best trade-off for typical indoor human motion at longer range (~5 m), preserving moving-edge structure while attenuating spurious/noisy events. Accordingly, we use *T_s_* = 20 ms for hand posture recognition and *T_s_* = 100 ms for human detection and pose estimation in this work.

**Figure 4:**
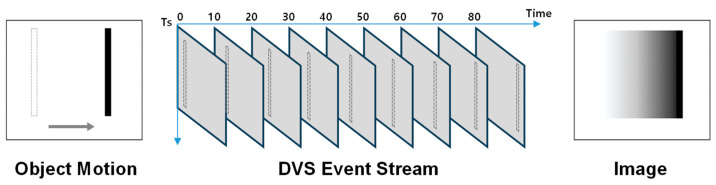
The polarity-agnostic timestamp-based image generation technique. Each pixel maintains the most recent event’s timestamp, and the timestamp difference *T* is mapped to an intensity *I*(*T*) using scaling parameters *T_s_* and *I_max_*, so that recently active edges become stronger while stale pixels fade. In this figure, we use *T_s_* = 100 ms (selected to best represent the event image under a typical indoor ~5 m setup and normal human walking speed) and *I_max_* = 255 for 8-bit grayscale intensity scaling.

With this mapping, pixels with recent events become bright (strong response), while pixels with older events gradually fade. In contrast to a raw/linearly normalized timestamp image (often used as a simple SAE visualization), the exponential decay concentrates dynamic range on recently active edges, producing clearer contours during intermittent motion. Importantly, because the timestamp memory is polarity-agnostic, the resulting image highlights edge presence independent of signed contrast, which is well matched to object detection and posture/pose recognition where edge shape is the primary cue. From a deployment perspective, the method requires only a single timestamp write per event and lightweight per-pixel mapping (implementable via a lookup table), keeping the overall procedure compatible with low-end edge processors. Figure 4 illustrates the conceptual flow of the proposed method. When an object moves, brightness changes occur mainly at its boundaries, and the DVS emits events at the corresponding pixels. The resulting event stream is accumulated only in the sense of updating a per-pixel last-timestamp memory (polarity is ignored). At a chosen sampling time, the stored timestamps are mapped to an intensity image via Equation (2), so that recently active edges appear bright while inactive/background pixels fade, yielding an image-form input for downstream inference.

To validate the proposed technique, we performed the action recognition task using the human activity dataset and DVS event simulator [17]. The public NTU RGB + D 120 human activity dataset includes a large-scale benchmark containing 120 action classes and 114,480 samples captured from 106 subjects [18]. The dataset provides synchronized multimodal streams including RGB, depth, infrared (IR), and 3D skeletons (25 body joints) recorded with three Microsoft Kinect v2 cameras. On the NTU RGB + D 120 dataset, we evaluated the proposed timestamp-based (recency) encoding while keeping the downstream recognition network and training protocol fixed in order to isolate the effect of the event image representation. As summarized in Table 1, the proposed encoding improves the overall accuracy from 0.896 (temporal accumulation) to 0.908. This improvement is consistent with the underlying signal characteristics of event streams in NTU120: for actions with slow, subtle, or intermittent motion, the event rate becomes sparse and temporally uneven, and simple accumulation over a window can yield ambiguous images (e.g., weak edges, fragmented contours, or mixed traces from temporally separated micro-motions). In contrast, the timestamp-based recency mapping emphasizes how recently each pixel was activated (rather than how many events were accumulated), which helps preserve the most recent moving-edge structure while suppressing stale residual activity within the window. Consequently, the proposed recency representation provides a more discriminative input for motion-centric recognition, particularly under low-motion or intermittent-event regimes that are common in fine-grained NTU120 actions. For context, the recent NTU120 action recognition literature (2023–2025) typically reports accuracies around 90–92% for strong skeleton-based methods, while multimodal approaches may reach into the low-to-mid 90% range with additional modalities and higher compute [19,20,21,22,23,24]. Because our Table 1 is designed to isolate the impact of encoding under a fixed downstream network (and uses event-simulated inputs), these numbers serve as a contextual benchmark level rather than a direct SOTA comparison.

## 4. Object Detection

This section focuses on system-level engineering optimizations required to meet real-time constraints in edge home surveillance deployments. We emphasize that the following techniques—such as channel/parameter reduction, pruning-related simplifications, stride manipulation, and quantization-aware choices—are standard practices in edge AI deployment and are not claimed to have standalone algorithmic novelty. Rather, they serve two purposes: (i) to demonstrate that the proposed event representation can be executed within strict latency/compute budgets, (ii) to provide reproducible implementation details for practitioners targeting similar edge devices and always-on monitoring scenarios.

**Figure 5:**
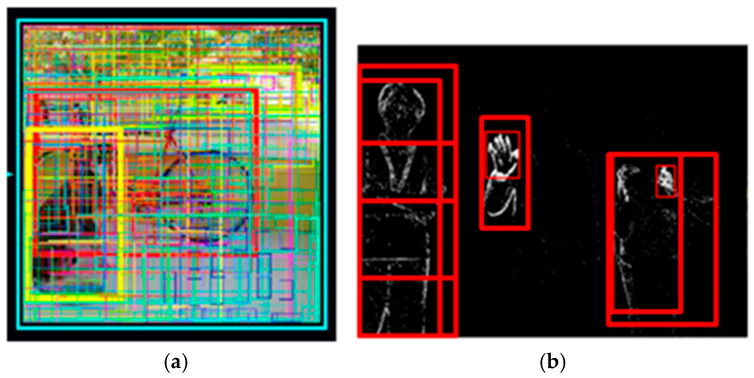
Comparison of RPN region proposals (proposal count overlay) for CIS vs. DVS. (**a**) CIS-based Faster R-CNN baseline visualizing the top N = 300 RPN proposals per image, following the standard setting reported in Faster R-CNN. (**b**) Event-based method using the DVS-specific detector described in the latter part of Section 4, where the average number of retained RPN proposals is ~9 per image, i.e., reduced by a few tens of times compared with (**a**), leading to a significantly lower RoI processing cost for edge deployment.

Privacy and user acceptance are primary constraints in in-home sensing. Prior studies on video-based in-home/assisted living monitoring report that the acceptance of conventional RGB cameras can be limited, with privacy concerns and perceived intrusiveness being major barriers particularly for intimate situations that may occur in private spaces [25]. Accordingly, we employ a DVS not only for computational efficiency but also as a privacy-aware sensing modality. Event representations have been discussed as a viable direction for privacy-preserving surveillance because they mainly encode moving boundaries of the subject while discarding much of the redundant visual content [26]. Object detection is required for home surveillance. Here, we employed DenseNet and Darknet architectures as feature extractors for human detection. The Faster R-CNN (FRCNN) structure gives the probabilistic location (region proposal) of humans. Figure 5 shows the region proposal results based on CIS and DVS images. The CIS image needs a huge amount of computations because it has many proposals due to background information while the DVS image includes only moving foreground objects, which in turn reduces the computational cost dramatically [27]. In FRCNN [28], the Region Proposal Network (RPN) generates candidate regions and the downstream RoI classification/regression cost scales with the number of retained proposals. Following the standard Faster R-CNN setting reported in the original paper (i.e., using *n* = 300 proposals per image while maintaining strong detection accuracy), we can visualize the top-300 RPN proposals for the CIS-based baseline in Figure 5a. Figure 5b uses the DVS-specific detector described in the latter part of Section 4 and shows that the average retained proposal count is ~9, i.e., reduced by a few tens of times. Since the primary motivation for using DVS in this work is edge deployment and model lightweighting, the proposal count reduction provides a clear quantitative explanation of why DVS is beneficial in our system.

**Figure 6:**
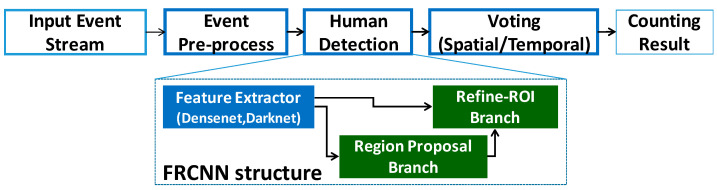
Algorithm flowchart of object detection based on DVS. The DVS event stream is converted into a frame-like event representation (timestamp-based image), then processed by a CNN backbone and a region proposal-based detection head to output human bounding boxes; the final detections are aggregated to provide a human counting result for applications such as crowd/occupancy detection.

Figure 6 shows the algorithm flowchart of object detection. The overall flowchart starts from the DVS sensing output and converts the event stream into a frame-like representation (e.g., the timestamp-based image described in Section 3), so that it can be processed by conventional CNN-based detection frameworks. After the event-derived image is generated, a backbone network (e.g., DenseNet/Darknet-based feature extractor) computes feature maps, and the detection head produces candidate regions (region proposals) followed by classification and bounding box regression to determine human locations. In this process, the DVS input typically contains fewer background-driven structures than CIS images (as observed in Figure 5), which helps reduce unnecessary proposals and improves computational efficiency for edge deployment. Finally, the proposed algorithm provides the human counting result by counting the number of detected human instances in the output, which can be directly used for practical applications such as crowd detection and occupancy monitoring.

We designed a computationally efficient network by iteratively shrinking the architecture and retraining after each change to ensure detection accuracy was preserved. Specifically, we reduced channel widths (up to quartering kernels) and removed convolutional layers, reverting to the previous configuration whenever performance degraded. To further lower computation, we used larger strides in the first two layers, which reduced feature map resolutions at later stages and thus decreased overall cost. This early downsampling also encourages a compact representation of the sparse event input, avoiding unnecessary computation on noisy fine details. In addition, we quantized both the weights and activations of convolutional layers into mixed-bit representation. More specifically, the weights and activations of the first convolutional layer were +1 and −1 [29]. For other residual convolutional layers, we employed a fixed-point implementation of 8-bit weights and activations [30]. As a result, we achieved a more than 11 times speed-up by using event-based processing, as shown in Table 2.

To contextualize compute and accuracy on modern event-based detection benchmarks, we reference RVT and its recent sparse attention variants SAST [31], which are representative state-of-the-art event-based detectors on Prophesee automotive datasets (e.g., Gen1 and 1Mp). In these benchmarks, the event sensor is mounted on a moving vehicle, so the input stream contains strong ego-motion and dense background events (roads, buildings, trees, signs), making foreground separation substantially more challenging than static camera indoor monitoring. SAST further reports backbone compute in terms of FLOPs and “A-FLOPs” (attention-related FLOPs) averaged over test samples, showing that RVT and SAST typically operate in the 0.8–2.2 G A-FLOPs regime (and higher when counting full backbone FLOPs, for example, the computational complexity of the detection head based on YOLOX [32] is 281.9 GFLOPs, as shown in Table 2), reflecting the need for large transformer-style backbones to achieve a high mAP under automotive ego-motion. The much smaller compute in our method (81 MFLOPs) is because the problem setting and design objective are different. First, our target scenario assumes a static DVS in indoor monitoring, where background is largely suppressed and the stream is dominated by edges induced by moving subjects; therefore, the detector primarily needs to recognize edge shape presence (e.g., person vs. non-person or presence counting) rather than solve full-scene, ego-motion-compensated multi-object detection. Second, we intentionally design an ultra-lightweight backbone (e.g., aggressive channel reduction, removing layers, and using larger early strides) to minimize edge compute, which directly reduces FLOPs by an order of magnitude compared with SOTA transformer backbones. Third, in our static camera setting the DVS naturally suppresses background, which reduces the number of candidate regions/proposals and contributes to a runtime reduction beyond what FLOPs alone predicts (we observe large end-to-end latency reductions together with compute reductions). In contrast, RVT/SAST are designed for automotive ego-motion benchmarks and aim at a high mAP under dense background events, which requires a substantially larger capacity and attention computation even after sparsity optimization.

**Figure 7:**
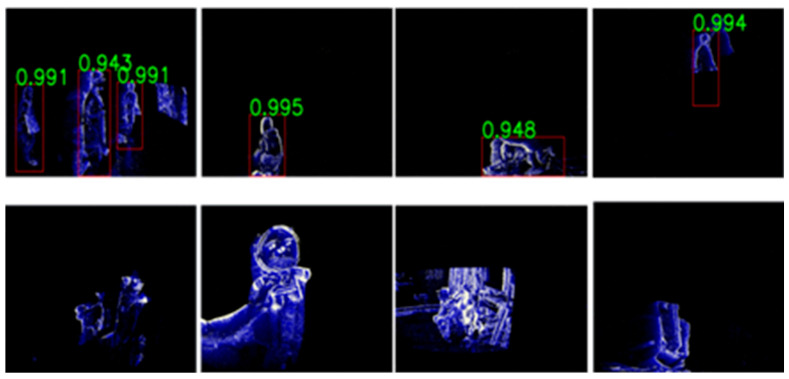
Sample images of the DVS training dataset for home surveillance. The dataset contains diverse positive human cases (various actions and occlusions, including overlapping) and hard negative indoor cases (e.g., pets and moving appliances), enabling robust detection with high recall (>95%) and low FAR (<2%). Our dataset was collected and labeled into three categories: human, animal, and others (e.g., household objects/background dynamics). The evaluation is performed on a held-out test set. In this work, we report accuracy on the test set as the probability of correctly predicting the human class. We define FAR using negative frames (frames labeled as non-human, i.e., animal or others) as the proportion of negatives incorrectly predicted as human.

The proposed algorithm is primarily designed for home surveillance occupancy detection, i.e., determining whether a person is present or absent in residential indoor environments. Accordingly, our experiments focus on static camera, indoor, human motion-centric scenarios, which reflect the intended deployment conditions (always-on operation, low latency, and edge compute constraints). In this context, for data generalization, we recorded DVS images across 10 home conditions (e.g., different rooms, illumination changes, daily activity patterns, and household layouts). Figure 7 shows representative sample images from the training dataset, which were collected to reflect realistic indoor conditions such as cluttered backgrounds, different distances to the sensor, and diverse human motions. The positive human dataset includes various ages, heights, genders, and clothing styles with diverse actions such as walking, jumping, duck-walking, crawling, and overlapping. This diversity is important because DVS images often contain sparse edge-like patterns rather than a textured appearance; therefore, robust detection requires the model to learn motion-driven body contours that may change significantly with posture, speed, and partial occlusions. In particular, overlapping and crawling cases are challenging for home surveillance because the visible edge structures can be fragmented and the scale/aspect ratio of the human region can vary rapidly. In addition, the negative dataset includes typical indoor objects and distractors such as chairs, curtains, TVs, dogs, cats, dolls, fans, and robotic vacuum cleaners. These negatives are intentionally included because many household objects can generate event responses (e.g., moving fan blades, robotic vacuum motion, or pet movement), which may otherwise cause false alarms. By training with such hard-negative examples, the detector can better distinguish true human motion patterns from non-human motion and background dynamics in a practical deployment setting. In total, we utilized 19.8 M DVS images for training. Specifically, we generated 19.8 M recency frames at 10 Hz from approximately 550 h of recordings collected across 10 rooms, three days, and eight participants. In addition, we constructed independent validation and test data (2 M DVS images in each dataset) collected under different home conditions (e.g., different rooms and/or different time periods) that was not used for training. While the proposed approach is effective for static camera indoor occupancy detection, its performance may degrade under conditions that violate the deployment assumptions, such as strong camera ego-motion, outdoor scenes with dense background events (e.g., wind-driven foliage, rain, strong illumination flicker), or tasks requiring fine-grained multi-class recognition across many object categories. Our method is therefore best interpreted as an edge-oriented representation and lightweight inference strategy for home surveillance. As a result, the recall accuracy and False Acceptance Rate (FAR) were measured to be >95% and <2%, respectively, indicating that the constructed dataset and training strategy are effective for reliable human detection under realistic smart-home scenarios. Under identical evaluation settings, we observe that the recall std is typically around ~0.5% and the false alarm rate (FAR) std is typically around ~0.1%. However, these statistical values are condition-dependent and should not be interpreted as universal constants. In home surveillance, the DVS event stream varies with ambient illumination and subject–sensor distance. As a result, both the mean performance and its dispersion change across environmental buckets. For example, when the illumination is ≥10 lux and the subject is within 5 m, recall is approximately 98%; under the same illumination, recall decreases to approximately 96% when the distance increases to 5–7 m. Under dimmer illumination (5–10 lux), recall is approximately 96% within 5 m, and further drops to approximately 92% at 5–7 m. Importantly, the std also differs across these buckets, reflecting different levels of event sparsity and edge contrast under varying illumination and range.

To benchmark the occupancy detection capability claimed in this paper, we use the performance of conventional Passive-Infrared-based (PIR-based) motion/occupancy sensors as a practical baseline for comparison. Importantly, PIR reliability is highly dependent on installation and environmental conditions; for example, reported presence detection accuracies can be as low as ~60% under typical ceiling placement and can improve to ~84% under more favorable placement, highlighting substantial variability in real deployments [33]. Moreover, long-term field testing in a single-family home reports an overall accuracy of 83.8% with a 12.8% false-positive rate (FPR) for commercial occupancy presence sensing, and explicitly identifies failure modes during prolonged static periods (e.g., sleep) [34]. Against this background, the high-recall and low-false-alarm operating points reported in this work (while satisfying strict edge compute/latency constraints) indicate that the proposed approach is a meaningful step beyond PIR-based baselines for occupancy-aware smart-home services.

## 5. Human Pose Estimation

Section 5 continues the deployment-oriented evaluation under edge constraints, reporting accuracy–latency trade-offs achievable with lightweight inference. The design choices presented here reflect practical engineering considerations (memory footprint, throughput, and real-time responsiveness) in home surveillance.

A pose estimation task can be used to control the home environment (i.e., illumination and temperature) and interact with a video game console. It has been recently reported that DVS images can be used for human pose estimation [35]. In practical edge AI scenarios, pose estimation is a useful semantic sensing function because it provides compact and actionable information (key-points) rather than full image content, which is well matched to the sparsity and motion-centric characteristic of DVS outputs. Here, we used the MS COCO 2017 key-point detection benchmark. The COCO key-point annotations define 17 anatomical key-points for each person. In our experiments, we adopted a top-down single-person setting, where each training sample corresponded to a single-person instance crop extracted from the COCO images (i.e., the multi-person images were converted into single-person training instances by cropping per annotated person). In terms of official COCO-2017 splits, the key-point task is based on train2017/val2017 [36]; for the key-point-labeled subset, commonly used splits include 56,599 training images and 2346 validation images with key-point annotations. In the top-down instance-based formulation, this corresponds to approximately 149,813 person instances for training and 6352 person instances for validation, where each person instance is treated as one single-person sample. Because our sensing modality was event-based, we converted the COCO single-person crops into synthetic event streams using an event simulator [17]. We evaluated pose estimation using the COCO key-point evaluation protocol based on Object Key-point Similarity (OKS), which plays a role analogous to IoU in detection. A predicted pose is matched to a ground-truth pose and considered correct if OKS ≥ 0.5. To design key-point estimation network models for pose recognition, we utilized HRNet because its performance is superior to other networks [36]. HRNet is effective in maintaining high-resolution representations and fusing multi-scale features, which generally improves the localization accuracy of body joints. However, because original HRNet has lots of redundant computations for processing DVS images, we pruned the backbone network to reduce the network size as described in Section 4. There is a trade-off between accuracy and computation. During the pruning procedure, we found that the number of stages and channels was strongly related to accuracy. For example, when the number of stages was reduced below three, accuracy dropped by 4%. Similarly, when the number of channels was halved, accuracy dropped by 14.7%. In all such compression steps, we fine-tuned the modified network after each stage/channel reduction, and we report the final post-fine-tuning performance (after accuracy recovery) for each configuration. These observations indicate that, although DVS inputs are sparse, pose estimation still requires a sufficient representational capacity to preserve fine spatial cues for joint localization. Therefore, rather than uniformly shrinking the model, we selectively reduced the redundant blocks such as high-resolution modules, ResNet blocks, branches, and connections in the fusion layer, while maintaining the essential stages and channel capacity that directly affect accuracy. Table 3 shows the comparisons between Vanilla HRNet [36] and the proposed lightweighted HRNet in terms of model size, accuracy, and processing time. As shown in Table 3, the proposed HRNet reduces the model size from 127 MB to 19 MB and improves processing time from 70 ms to 6 ms on NVIDIA Titan X, while maintaining a comparable pose estimation accuracy (0.95 to 0.94). We confirmed that we could achieve more than an 11× speed-up by using the event-based processing while maintaining the accuracy. Recent frame-based 2D human pose estimation commonly adopts HRNet-W48 as a strong baseline, and performance improvements are often achieved by either increasing model capacity or using multiple networks during training/inference. In particular, DE-HRNet [37] enhances HRNet-style high-resolution features by introducing detail enhancement components and reports a modest gain on COCO test-dev (384 × 288); compared with HRNet-W48 (AP 0.755 with 63.6 M parameters), DE-HRNet-W48 reports AP 0.757 while increasing the backbone size and compute to 74.8 M parameters (which corresponds to an FP16 checkpoint size increase from roughly 127 MB to 150 MB). In a different direction, Boosting Semi-Supervised 2D HPE [38] improves accuracy primarily via stronger semi-supervised training and, for a higher performance, adopts a dual-network setting (i.e., two identical yet independent networks). In their COCO test-dev comparison, the dual-network configuration effectively doubles model capacity relative to a single HRNet-W48 backbone and achieves an improved accuracy (e.g., 0.952 @AR OKS ≥ 0.5 in a dual setting). In contrast, our work targets event-based edge deployments (e.g., static camera indoor monitoring) and focuses on improving the event-to-image representation (polarity-agnostic recency encoding) so that a competitive accuracy and real-time operation can be achieved without scaling up the downstream network. In other words, while benchmarks exemplify the common strategy of improving COCO pose accuracy by increasing backbone capacity or the number of networks, our approach emphasizes representation-level efficiency tailored to DVS streams and edge constraints.

## 6. Hand Posture Recognition

**Figure 8:**
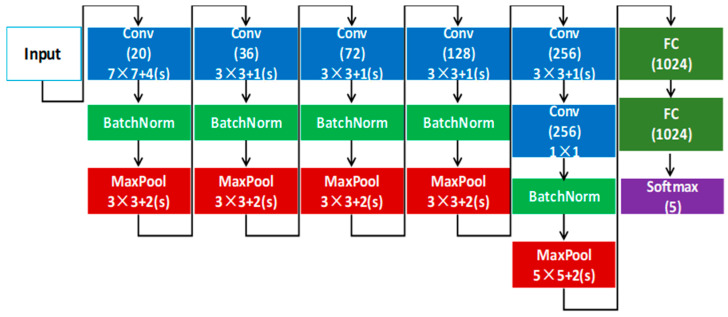
Network structure for low-latency posture recognition based on DVS. The proposed model uses five convolutional layers to extract posture-related features from sparse event-derived images and two fully connected layers for final classification, enabling reliable HMI operation with high recall (99.19%), low FAR (0.0926%), and real-time latency (14.31 ms on i5-4590 single core). We use the notation ‘k × k + (s)’ to indicate kernel size and stride for each convolution/pooling layer.

The proposed event-based processing technique can be used for another edge AI task. For example, hand posture recognition is required for HMI at edge devices. In practical smart-home and wearable scenarios, hand posture recognition can provide a direct and intuitive control signal, enabling natural interaction without requiring high-resolution texture information. This is well aligned with the characteristics of DVS, because DVS mainly captures motion-driven edges and suppresses static background, which can reduce irrelevant visual content and allow the model to focus on dynamic hand contours. In our previous work [39], we proposed a low-latency optical flow and gesture estimation algorithm. While optical flow-based approaches are effective for dynamic gestures, real edge deployments often benefit from a compact posture classifier that can operate reliably even when (i) motion is intermittent, (ii) the hand moves at different speeds, (iii) only partial edges are available due to viewpoint changes or occlusions. Specifically, optical flow-based methods inherently face the aperture problem, where local edge motion constrains only the normal component, requiring spatial/temporal regularization that can be sensitive to spurious events, viewpoint changes, and mechanical vibration—factors that may increase false triggers in always-on HMI settings. In contrast, the present work intentionally adopts a compact posture/edge-shape classifier (rather than a velocity field estimator) and evaluates reliability using sequence-level FAR with majority voting over a short temporal window. This posture-centric formulation reduces the dependence on stable flow estimation and thus is better aligned with HMI requirements where low false acceptance is as critical as accuracy. While many event camera gesture recognition studies primarily report classification accuracy (and often omit an explicit FAR definition under negative sequences [40]), we explicitly report FAR for system-level reliability in practical deployment. To obtain a reliable HMI performance, we designed a lightweight CNN-based posture recognition network that directly processes DVS-derived representations and outputs posture labels with minimal computation. In our HMI experiment, the posture classification task is intentionally simple with three posture categories (Rock–Paper–Scissors), which contributes to a low FAR under the tested conditions. Leveraging the sparsity and binary nature of DVS images, we designed a low-latency CNN classifier as shown in Figure 8. The convolutional stack extracts hierarchical edge/motion features from the sparse inputs, and the fully connected layers perform compact classification. Following the ConvNet-based motion recognition architecture in our prior work which employs five convolutional layers and three fully connected layers [12], we started from an analogous lightweight classifier for the HMI task and performed an ablation study to minimize computation while preserving reliability. In particular, we systematically varied the kernel size and stride of the early convolution/pooling stages, because these parameters dominate the spatial downsampling rate, feature map size, and thus overall FLOPs. After each architectural change, the network was fine-tuned and evaluated on the held-out test set; only configurations that maintained recall accuracy and FAR at the same level as the reference configuration were retained. This ablation-driven procedure resulted in the final compact design shown in Figure 8, achieving a substantially reduced computational cost. We implemented the proposed algorithm on a low-end PC to validate feasibility under realistic edge constraints. The measured recall and False Acceptance Rate (FAR) were 99.19% and 0.0926%, respectively. We measured FAR at the sequence level (not per-frame). Each test sample corresponds to a 10-frame gesture sequence, and the classifier outputs per-frame confidence scores that are aggregated into a single sequence-level decision using majority voting. A sequence is accepted only when the maximum class confidence exceeds a predefined threshold. We define false acceptance as the case where a non-target (negative) sequence is incorrectly accepted as one of the gesture commands. Accordingly, FAR is computed as FAR = N_FP_/N_neg_ × 100 (%), where N_FP_ is the number of non-matching (ground-truth ≠ predicted) sequences that are accepted and N_neg_ is the total number of negative sequences in the test set. Specifically, each decision is made from a 10-frame sequence, and we set a voting threshold over these 10 frames to minimize FAR (i.e., a gesture is accepted only if sufficient per-frame votes/support are accumulated within the 10-frame window). These results indicate that the proposed approach can provide both a high detection sensitivity (high recall) and strong robustness against false triggers (low FAR), which are important requirements for HMI applications where user experience and safety depend on stable recognition outputs. In addition, we confirmed that the overall latency was measured to be 14.31 ms (@i5-4590 CPU, single core), which was enough to be used for real-time interaction even in a low-end processor. This low latency suggests that the proposed event-based posture recognition can be integrated into always-on edge devices without requiring GPUs or high-end NPUs, and it can be combined with other event-driven perception modules (e.g., detection and pose estimation) to build a complete low-power interactive vision system.

## 7. Discussions and Conclusions

We have proposed and demonstrated event-based machine vision for edge AI computing. In contrast to conventional frame-based sensing, the proposed event-based approach leverages the activity-driven nature of a DVS and designs low-latency vision algorithms that exploit sparsity in both sensing and computation. As a result, the proposed framework provides a practical pathway to deploy reliable real-time perception modules under tight constraints of bandwidth, memory, and power at the edge, where always-on operation is often required. A major advantage of event-based sensing is that it can drastically reduce the end-to-end data burden in long-duration monitoring scenarios. Under the same indoor monitoring condition (640 × 480 resolution, 24 h recording with intermittent human motion), the recorded raw DVS stream becomes about 30 times smaller than conventional CIS video, which directly alleviates interface throughput, memory bandwidth, and storage requirements. Importantly, the benefit remains meaningful even after compression: the compressed DVS data size is still about five times smaller than CIS, implying that sparsity is advantageous not only before compression but also after standard storage pipelines. From a deployment perspective, such reductions translate to tangible cost and scalability advantages by lowering both CAPEX/OPEX related to continuous recording, data transfer, and storage management in real smart-home/building environments. In addition to data efficiency, we demonstrated that event-to-image conversion can be improved in a way that is well matched to edge AI inference. Specifically, the timestamp-based encoding provides a frame-like representation that preserves the recency of activity, which is particularly important in indoor scenes where motion is intermittent and the event stream can be sparse. In the action recognition validation, the proposed timestamp-based encoding showed a higher accuracy than conventional temporal accumulation (0.908 vs. 0.896), indicating that recency-aware encoding can improve downstream recognition robustness without increasing the data volume. We also verified that event-based processing enables substantial computational acceleration while maintaining practical recognition quality. By designing a computationally efficient detection method (including architectural simplification and mixed-precision quantization), the event-based implementation achieved more than 11× speed-up, reducing processing time from 172 ms to 15 ms and decreasing FLOPs from 5.8 G to 81 M in the reported comparison setting. For pose estimation, a lightweight HRNet configuration further reduced the model size (127 MB → 19 MB) and inference time (70 ms → 6 ms) while keeping comparable mAP (0.95 → 0.94), supporting the feasibility of real-time pose estimation with a compact network for edge deployment. Furthermore, for hand posture recognition as an HMI-oriented edge task, the proposed low-latency model achieved high recall (99.19%), low FAR (0.0926%), and real-time latency (14.31 ms) on a low-end CPU, demonstrating that responsive interaction can be supported even without high-end accelerators. Overall, these results collectively suggest that event-based machine vision is not only a sensor-level novelty but also a system-level opportunity: (i) it reduces the data footprint for continuous monitoring, (ii) it enables efficient representations suitable for CNN-based techniques, (iii) it unlocks significant latency and compute savings that are critical for always-on edge AI devices. Finally, as image sensors become even more widespread with the ubiquitous IoT and AR/VR glasses, computationally efficient visual processing systems will play a critical role in enabling privacy-aware, real-time, and scalable edge perception, and event-based sensing provides a strong foundation for such next-generation applications [41,42,43,44].

A known limitation of event-based sensing is that perfectly stationary objects may generate few or no new events, which can reduce instantaneous evidence for purely event-driven detection during long static periods. This motion–static trade-off is inherent to DVS sensing and is particularly relevant to surveillance scenarios where an intruder might remain motionless. However, our target application is home surveillance occupancy detection (person present/absent) rather than fine-grained static recognition. In such a system, robustness to long static periods can be strengthened by combining object detection with a lightweight tracking module [45] operating on the detected bounding boxes. Specifically, once a person is detected, a tracker can maintain and update the target state over time and distinguish among three practically important cases: (i) the target leaves the field of view (track termination near image boundaries or consistent outward motion), (ii) the target enters the field of view (track initialization with inward motion), (iii) the target remains in the scene with little or no motion (a persistent track with minimal displacement and low event rate). These considerations highlight that, while DVS sensing is intrinsically motion-driven, a practical occupancy detection system can explicitly handle static intervals through tracking-based state maintenance without sacrificing the low-latency, edge-efficient nature of the proposed technique. As future work, we plan to build and evaluate a complete end-to-end home surveillance system that integrates the proposed DVS-based detection with a lightweight bounding box tracking module, enabling more robust state reasoning (enter/leave/static) under long static periods and slow-motion scenarios.

## Figures and Tables

**Table 1:** Performance comparison between the previous (temporal accumulation) and proposed (timestamp-based encoding) image generation techniques. The timestamp-based (polarity-agnostic recency) encoding shows a higher accuracy (90.8%) than temporal accumulation (89.6%) on the evaluated human activity dataset setting, indicating the benefit of recency-aware encoding for event-to-image conversion.

Data Set	Image Generation	Accuracy
NTU RGB + D 120	Temporal accumulation	89.6%
	Timestamp-based encoding	90.8%

**Table 2:** Computation comparison between conventional and event-based vision processing.

FRCNN + FPN	Number of Layers ^1^	FLOPs	Accuracy	Processing Time (ms)
Conventional	91	5.8 G ^2^	0.96 ^3^	172 ^2^
Event-based	24	81 M ^2^	0.95 ^3^	15 ^2^
RVT [31]	YOLOX ^4^ [32]	281.9 G ^5^	0.512 ^6^	17.3 ^5^

^1^ Backbone network, ^2^ computed on Titan X, ^3^ recall accuracy, ^4^ detection head, ^5^ computed on Tesla V100, ^6^ measured AP on COCO dataset.

**Table 3:** Comparisons between the Vanilla HRNet and the proposed lightweight HRNet for DVS-based pose estimation. The table reports model size, accuracy, and inference latency measured on NVIDIA Titan X. The proposed HRNet substantially reduces the model size (127 MB → 19 MB) and processing time (70 ms → 6 ms) while maintaining a comparable accuracy with only a minor decrease (0.95 → 0.94), demonstrating that pruning redundant modules and connections is effective for real-time edge deployment.

Model	Size	Accuracy	Processing Time @NVIDIA Titan X
Vanilla HRNet	127 MB	0.95	70 ms
Proposed HRNet	19 MB	0.94	6 ms
Boosting Semi-Supervised 2D HPE	254 MB	0.952	~140 ms ^1^

^1^ Estimated from the model size [38].

## Data Availability

Third-party data. Restrictions apply to the availability of these data.

## Abbreviations

The following abbreviations are used in this manuscript:

AIoT	Artificial Intelligence of Things
CMOS	Complementary Metal–Oxide–Semiconductor
CIS	CMOS Image Sensor
DVS	Dynamic Vision Sensor
HMI	Human–Machine Interface
CAPEX	Capital Expenditure
OPEX	Operating Expenditure
RGB	Red Green Blue
CNN	Convolutional Neural Network
RPN	Region Proposal Network
RoI	Region of Interest
AP	Average Precision
FAR	False Acceptance Rate
FLOPs	Floating Point Operations Per Second
AR	Augmented Reality
VR	Virtual Reality
